# Metformin and arsenic trioxide synergize to trigger Parkin/pink1-dependent mitophagic cell death in human cervical cancer HeLa cells

**DOI:** 10.7150/jca.61299

**Published:** 2021-08-28

**Authors:** Jing Chen, Cunmin Zhou, Juan Yi, Jingjing Sun, Bei Xie, Zhewen Zhang, Qunfeng Wang, Gang Chen, Suya Jin, Jinxia Hou, Miao Qin, Lina Wang, Hulai Wei

**Affiliations:** 1 Key Laboratory of Preclinical Study for New Drugs of Gansu Province, School of Basic Medical Sciences, Lanzhou University, Lanzhou, Gansu, China.; 2 The first hospital of Lanzhou University, Lanzhou, Gansu, China.

**Keywords:** Metformin, Arsenic Trioxide, Mitophagy, Pink1/Parkin, HeLa

## Abstract

Mitochondria are involved in various biological processes including intracellular homeostasis, proliferation, senescence, and death, and mitochondrial mitophagy is closely related to the development and regression of malignant tumors. Recent studies confirmed that the hypoglycemic drug metformin (Met) exerted various antitumor effects, protected neural cells, and improved immunity, while arsenic trioxide (ATO) is an effective chemotherapeutic agent for the clinical treatment of leukemia and various solid tumors. However, the possible combined antitumor effects of Met and ATO and their cellular molecular mechanisms are unclear. We investigated the role of Parkin-mediated mitochondrial mitophagy in the anti-tumor mechanism of Met and ATO by studying the effects of Met and/or ATO on the proliferation and apoptosis of cervical cancer HeLa cells. Both Met and ATO effectively inhibited the proliferative activity of HeLa cells and induced apoptosis by activating Bax and inhibiting Bcl-2. Met and ATO treatment alone or in combination stimulated mitophagosome accumulation in HeLa cells, increased the conversion of microtubule-associated protein light chain 3 (LC3)-I to LC3-II, and decreased levels of the mitophagic lysosomal substrate protein P62. The mitochondrial membrane potential of HeLa cells also decreased, accompanied by activation of the mitochondrial translocase TOM system and the Pink1/Parkin signaling pathway. These results suggested that Met and/or ATO could induce mitophagy in HeLa cells via the Pink1/Parkin signaling pathway, leading to mitophagic apoptosis and inhibition of tumor cell proliferation. The combination of Met and ATO thus has enhanced antitumor effects, suggesting that this combination has potential clinical applications for the treatment of cervical cancer and other tumors.

## Introduction

Numerous recent studies have suggested that, in addition to its hypoglycemic effect, metformin (Met) can reduce the incidence of malignant tumors and inhibit the growth of tumor cells. The potential antitumor effect of Met is believed to be independent of its hypoglycemic effect 1-5, making Met a hot topic in the field of antitumor research. However, its antitumor properties and mechanism remain unclear. Sacco *et al.* used high-resolution mass spectrometry to analyze the effects of Met on the proteome and phosphorylated proteome in breast cancer cells, and demonstrated that Met enhanced the sensitivity of breast cancer cells to apoptosis-inducing agents and decreased their sensitivity to proliferative substances 6. Cytological studies further confirmed that Met significantly inhibited cell growth and proliferation, and induced apoptosis in breast, prostate, pancreatic, lung, and liver tumor cells 6-11. However, the anti-tumor mechanism of Met is complicated. Met phosphorylates and activates AMP-activated protein kinase (AMPK), which in turn inhibits mammalian target of rapamycin (mTOR) complex 1 (mTORC1) and mTORC2 pathways and downstream signaling molecules, regulates cell growth signaling pathways related to tumor proliferation and apoptosis, and inhibits tumor proliferation. Met also exerts anti-tumor effects through activation of AMPK, while p53 can be directly phosphorylated and activated by AMPK, playing important roles in cell cycle arrest, induction of apoptosis, and mitophagy 12. Met decreases the phosphorylation of phosphoinositide 3-kinase and Akt, which in turn inhibits the mTOR signaling pathway, suppresses abnormal proliferation, and reduces the apoptosis tolerance of tumor cells. Met can inhibit microangiogenesis and reduce microvessel density through activation of the AMPK/mTOR pathway and inhibit the progression of ovarian cancer 11,13,14. Met can also inhibit the invasion and metastasis of tumor cells by suppressing the epidermal-mesenchymal transition, and plays a role in activating the anti-tumor immune response and anti-tumor stem cells 2,15,16.

Inhibition of mitochondrial activity or disrupted mitochondrial function blocks the energy required for tumor cell growth, leading to inhibition of their proliferation and growth 17. Mitochondria also play a key role in apoptosis and mitophagy 18,19. Met was shown to activate the liver kinase B1/AMPK pathway by disrupting the activity of mitochondrial respiratory chain complex I, inhibiting oxidative phosphorylation in mitochondria, and increasing the intracellular AMP/ATP ratio 20. Met inhibited the proliferation and growth of breast cancer cells by inhibiting mitochondrial activity and causing dysfunction or alteration of mitochondrial function 5. Met was also shown to induce mitophagy in tumor cells 3, but this appears to contradict the action of Met in tumor cells. Moreover, Met reduces inflammation associated with senescence in normal T cells by enhancing mitophagy and normalizing mitochondrial function, which in turn delays cellular senescence. However, the role of Met-induced cellular mitophagy, especially mitochondrial mitophagy, in its anti-tumor function, has not been demonstrated.

Met has synergistic effects with various chemotherapeutic agents and targeted drugs in the antitumor field 21,22. Met inhibited the gene expression of *MDR1* and P-gp in tumor cells, thereby increasing their sensitivity to tumor chemotherapeutic agents 23. Met also enhanced the antitumor effects of other chemotherapeutic agents by inhibiting insulin/insulin-like growth factor-I signaling, improving the sensitivity of crizotinib-sensitive and drug-resistant non-small cell lung cancer, inhibiting cell proliferation, reducing invasive capacity, and promoting apoptosis 24,25. However, many questions remain about the effects of Met-enhancement on other antitumor drugs and their molecular mechanisms. Arsenic trioxide (ATO) shows promise for the treatment of leukemia and various other solid tumors 26,27. Studies confirmed that ATO induced tumor cell differentiation, inhibited tumor cell proliferation, and induced apoptosis, as well as inducing cellular autophagy while inducing apoptosis in tumor cells 28. However, the potential synergistic effects of Met and ATO in inducing tumor cell death and mitochondrial apoptosis via a mitochondrial mechanism remain unknown.

Cervical cancer is the forth-leading killer of women 29, with rates increasing and patient age decreasing annually in recent years. The main treatments for cervical cancer are surgery in the early stage, and radiotherapy/chemotherapy in the middle and late-stages. In view of the current limitations of cervical cancer treatment, there is an urgent scientific and practical need to explore the possible effects and mechanisms of Met and/or its combination with ATO in cervical cancer cells. We therefore examined the synergistic effects of ATO and Met on the proliferation, apoptosis, and autophagic activity of cervical cancer HeLa cells, and investigated if Parkin-mediated mitochondrial mitophagy played a key role in the anti-tumor effect of Met alone and in combination with ATO.

## Materials and Methods

### Main reagents

Dulbecco's Modified Eagle Medium was obtained from Gibco BRL (Maryland, USA), fetal bovine serum was from BI Biotechnology (Kibbutz Beit Haemek, Israel), and TRIzol reagent was purchased from Invitrogen (Penrose, Auckland, New Zealand). RIPA protein lysate and BCA protein concentration assay kits were from Solebro (Beijing, China), rabbit anti-microtubule-associated protein light chain 3 (LC3) polyclonal antibody, rabbit anti-Beclin1 polyclonal antibody, rabbit anti-p62 polyclonal antibody, TOM20, Caspase-3, and PINK1 were all purchased from Cell Signaling Technology (Boston, USA). Green fluorescent protein (GFP) and color pre-stained marker were from Thermo Scientific (Massachusetts, USA), mouse anti-human β-actin antibody was from Biovision, Inc. (California, USA), and ATO and Met were purchased from Sigma-Aldrich (Merck KGaA, Darmstadt, Germany).

### Cell culture

Human cervical cancer HeLa cells, HeLa-GFP-LC3 cells with GFP-LC3 gene, and LC3-GFP-Parkin cells with GFP-Parkin gene (*PRKN*) were provided by Professor H.M. Shen's laboratory at the National University of Singapore and stored in the Laboratory of Medical Laboratory Animal Sciences, School of Basic Medical Sciences, Lanzhou University. The cells were cultured in complete culture medium containing 10% fetal bovine serum and 5% CO_2_, and routinely cultured at 37°C.

### MTT assay to detect cell proliferation

The cells were inoculated at 0.8 × 10^5^ cells/mL in a 96-well culture plate. Different concentrations of the test drugs were added and the cells were incubated at 37°C with 5% CO_2_ for 24-72 h. At 4 h before termination of the culture, 10 μl of MTT (Solebro Life Sciences, Beijing, China) solution (5 mg/mL) was added and the culture was continued for 4 h. Sodium dodecyl sulfate (SDS) 10% was then added to the wells overnight at 37°C. The absorbance (optical density) of each well was measured at 570 nm using a spectroscopic multiplate reader (Powerwave X, Bio-Tek, Vermont, USA), and the cell proliferation inhibition rate was calculated relative to the control.

### Annexin V-PE/7-AAD assay for apoptosis detection

Cells were collected, washed with phosphate-buffered saline (PBS) and resuspended in 500 μl binding buffer, followed by adding 5 μl fluorescein isothiocyanate-labeled Annexin V-PE and 5 μl 7-AAD and staining for 30 min at room temperature in the dark. Apoptosis of the treated cells was determined by flow cytometry.

### Western blotting analysis of protein expression

The target cells were collected and washed with PBS. Total proteins were extracted from RIPA lysate and further quantified by the BCA method refer to the instruction. The proteins were separated by 10% SDS-polyacrylamide gel electrophoresis and transferred to a polyvinylidene difluoride membrane. The membrane was incubated with 5% skimmed milk powder at room temperature for 1 h to block potential non-specific binding. The corresponding primary antibodies were added separately and incubated overnight at 4°C. The membrane was washed three times with PBS-T for 5 min each. Horseradish peroxidase-labeled secondary antibodies were then added and incubated at room temperature for 1 h. After washing three times with PBS-T, the membrane was developed using ultrasensitive enhanced chemiluminescent (Millipore, Massachusetts, USA) solution (liquid A: liquid B=1:1) and placed on a chemiluminescent analyzer (WD-9423C, Beijing Six One Biotechnology Co., Ltd. China) for scanning imaging and analysis.

### Cell morphology observation

#### Phase contrast microscopy

Cells in logarithmic growth stage were inoculated into cell culture flasks at 0.5 × 10^5^ cells/mL, 6 ml per flask. After reaching cell confluence, different concentrations of Met and ATO were added and the cell morphology was observed under an inverted light microscope after 24 h of incubation.

#### Wright-Giemsa staining detection

Clean sterile coverslips were placed in a 6-well plate and 0.5 × 10^5^ cells were inoculated in each well. After the cells attached to the coverslips, Met and ATO were added into the wells at different concentrations. The coverslips were then removed after 24 h of incubation, fixed in methanol for 30 min, and stained with Wright-Giemsa staining solution for 10-15 min.

#### Co-localization of mitochondria and autophagosomes by laser confocal microscopy

GFP-LC3-HeLa cells were collected and inoculated in 6-well plates at a density of 0.5 × 10^5^ cells/mL. The cells were incubated for 24 h followed by the addition of ATO and Met and incubation for a further 6 h. Mito tracker red (Thermo Fisher Scientific, Massachusetts, USA), 1 mmol/L was then added to each well to a final concentration of 500 nmol/L and incubated at 37°C for 30 min. The distributions of red and green fluorescence in the cells were observed by laser confocal microscopy.

#### Transmission electron microscopy

The target cells were collected, washed with PBS, and fixed with 3% glutaraldehyde overnight at 4°C. The fixed cells were washed with PBS again, placed in 50% ethanol for 10 min, 70% ethanol for 10 min, 90% ethanol for 10 min, 100% ethanol for 10 min, and acetone for 10 min. The cells were further mixed with embedding agent and acetone solution at a ratio 1:1 and then soaked at room temperature for 2 h. After embedding in epoxy resin, the cells were incubated at 35°C for 12 h, 45°C for 24 h, and 65°C for 24 h, and the blocks were sliced and stained with uranyl acetate and lead citrate. The ultrastructure of the cells was then observed under transmission electron microscope.

### Statistical analysis

All data are expressed as the mean ± standard deviation and were analyzed using SPSS 18.0 statistical software (IBM, Chicago, USA). Differences between groups were compared by the Newman-Keuls *post hoc* test, and differences between two samples within groups were compared using Student's *t*-test.

## Results

### Met combined with ATO to inhibit proliferation of HeLa cells

**Figure 1:**
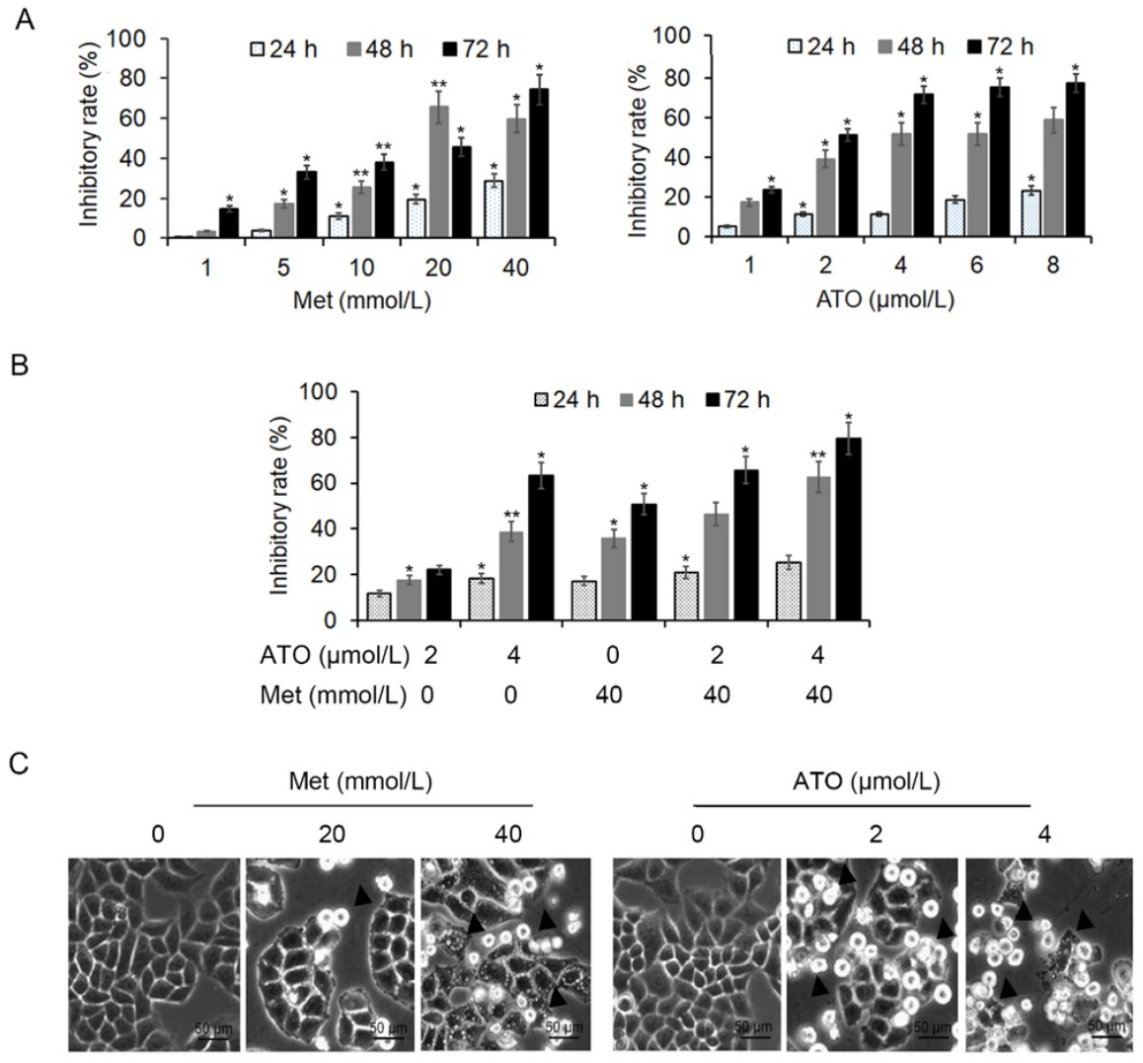
Inhibition of proliferation activity of HeLa cells by Met and ATO. Effects of Met and ATO on proliferative activity of HeLa cells. (A) Proliferation inhibition rate of HeLa cells after treatment with Met and ATO, respectively, (B) and with Met and ATO combined. (C) Changes in cell morphology under phase contrast microscopy after Met and ATO treatment of HeLa cells for 24 h (as indicated by ▲) (original magnification, 20×). *P < 0.05 and **P < 0.01 compared with control group.

HeLa cells were treated with 20 mmol/L and 40 mmol/L Met and 2 μmol/L and 4 μmol/L ATO, either alone or in combination, for 24 h. MTT colorimetric assay showed that both Met and ATO inhibited the proliferation of HeLa cells in a concentration-time-dependent manner, and the effect was more pronounced following combined addition of the two drugs (Figure 1A and B). Numerous cells were shed from the flask wall and floated in the medium, as seen by light microscopy, with higher concentrations of Met/ATO associated with more shed cells. Adherent cells in the high-concentration group were wrinkled and the refractive property of the cell membrane was reduced (Figure 1C).

### Met enhanced ATO to induce apoptosis in HeLa cells

#### Changes in cell morphology

**Figure 2:**
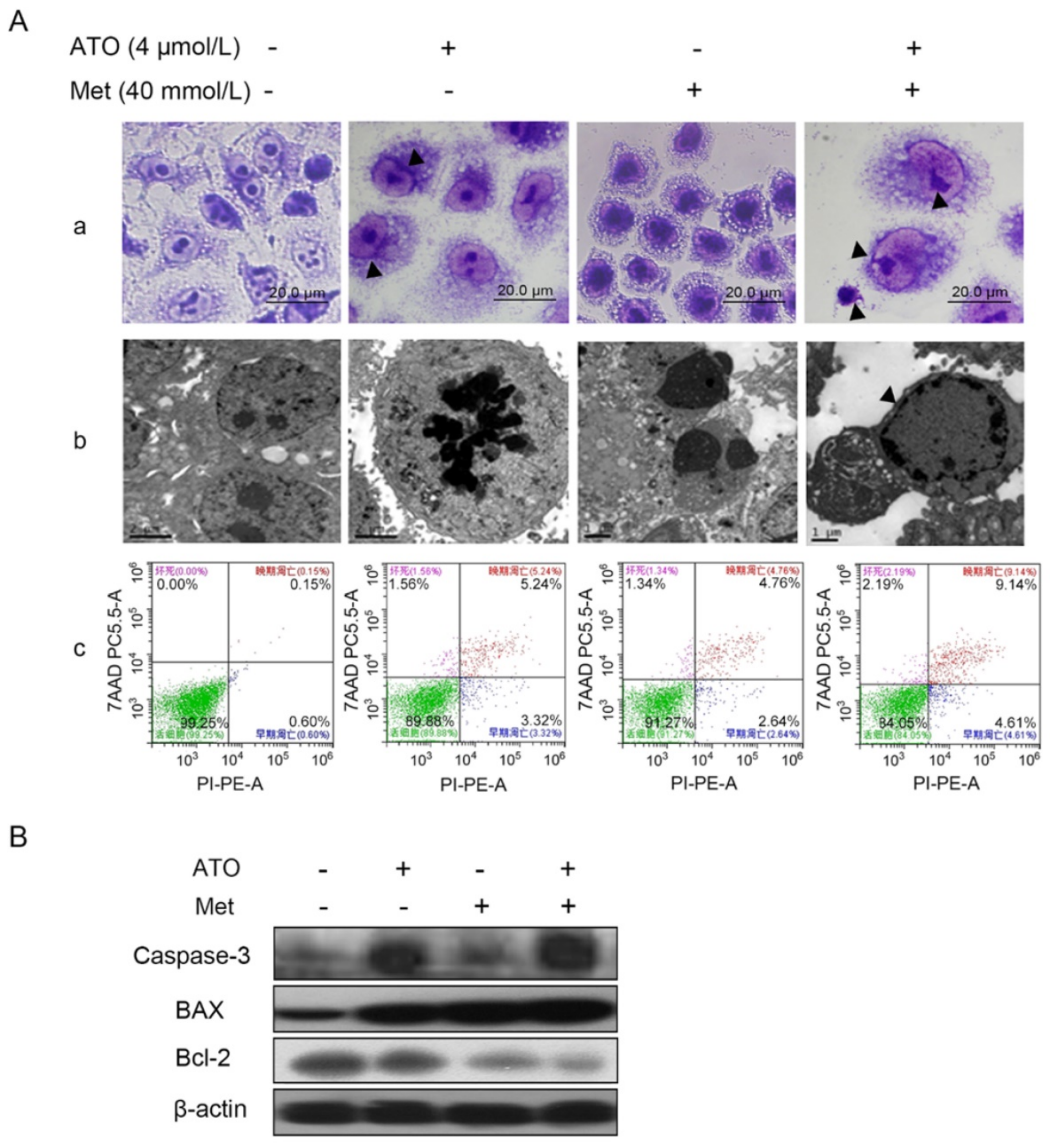
Met promotes the apoptosis-inducing effect of ATO on HeLa cells. (A) Changes in cell morphology were observed by light miscroscopy after treatment of HeLa cells with 4 μmol/L ATO and 40 mmol/L Met, alone or in combination, for 24 h, shown by Wright-Giemsa staining (as pointed to by ▲) (original magnification, 100×). (a) Cell ultrastructure was examinated by transmission electron microscopy. (b) Apoptosis was detected by flow cytometry after Annexin V-PE/7-AAD double staining and apoptosis rates were compared (c). (B) Changes in apoptosis-related proteins.

HeLa cells treated with 40 mmol/L Met and 4 µmol/L ATO, alone or in combination, were observed by light microscopy after Wright-Giemsa staining. The cells showed apoptotic morphological changes including dense deep staining of cytoplasm and the chromatin border in the nucleus (Figure 2A-a). The cells also presented typical apoptotic morphological changes including vacuolar degeneration, dense chromatin, edge set, nuclear fragmentation, and apoptotic vesicle formation under transmission electron microscopy (Figure 2A-b). Flow cytometry showed that the apoptosis rate of HeLa cells increased after Met or ATO treatment, and especially after combined treatment (Figure 2A-c). These results suggested that ATO could promote the apoptosis-inducing effect of Met on HeLa cells.

Bcl-2 family proteins are key molecules in the regulation of apoptosis, and can act directly or indirectly on mitochondria to perform their corresponding functions 30. Compared with treatment of HeLa cells with Met or ATO alone, the combination of 40 mmol/L Met and 4 µmol/L ATO resulted in significant downregulation of the anti-apoptotic protein Bcl-2, significant upregulation of the pro-apoptotic protein Bax, and an increased Bax/Bcl-2 ratio (Figure 2B). The apoptosis effector and actuator caspase-3 was also significantly upregulated, suggesting that both Met and ATO significantly inhibited the proliferation and induced apoptosis of HeLa cells, and the combination of Met and ATO had a synergistic effect.

### Met synergized ATO to induce mitophagy and trigger mitophagic cell death in HeLa cells

**Figure 3:**
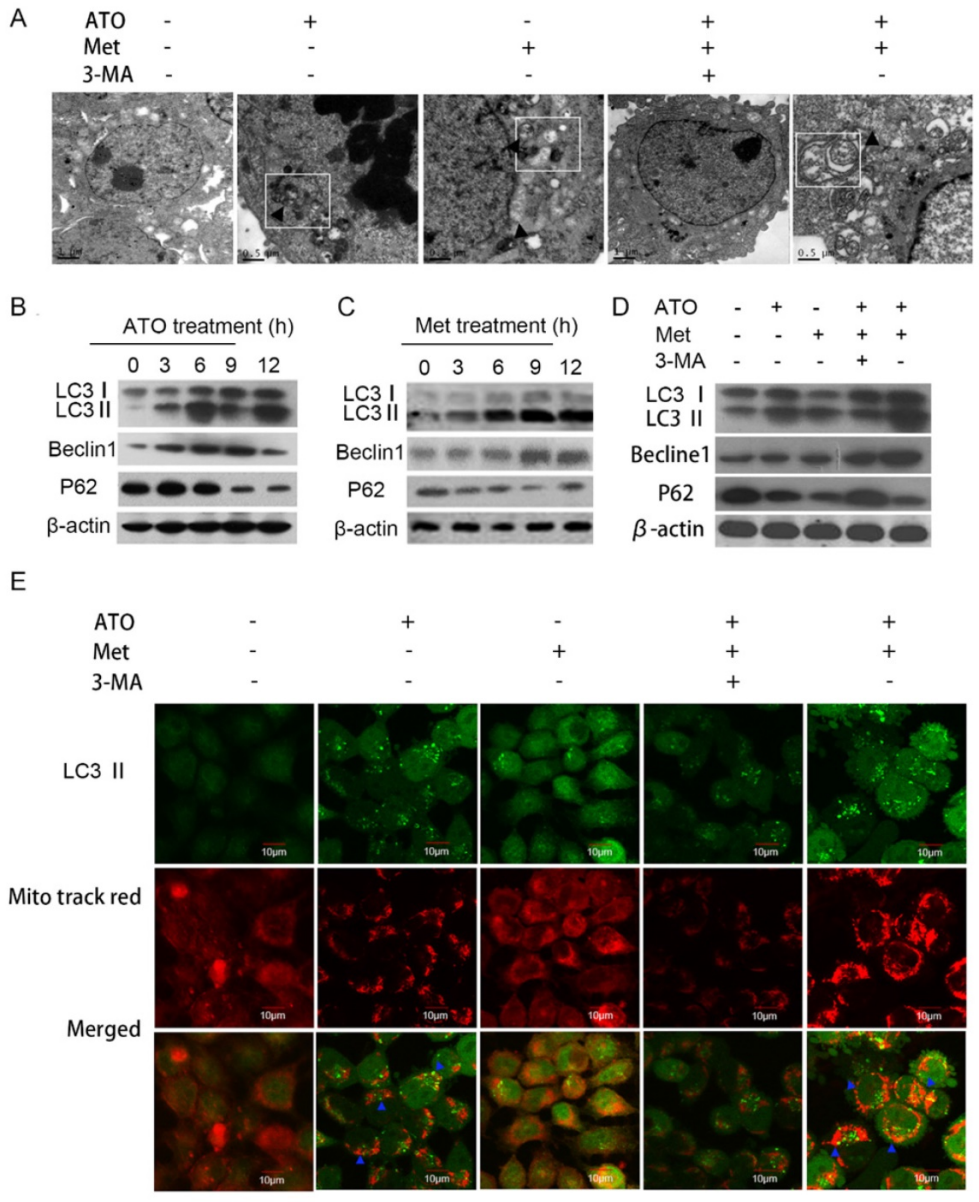
ATO and Met induced autophagy and mitochondrial autophagy in HeLa cells. (A) Morphology of autophagic vesicles and mitochondrial autophagosomes in HeLa cells by transmission electron microscopy (as showed by ▲ in □). Intracellular LC3-II, Beclin1, and p62 protein levels in HeLa cells treated with 4 µmol/L ATO for 0-12 h (B), or 40 mmol/L Met for 0-12 h (C). (D) Changes in levels of LC3-II, Beclin1, and p62 after treatment with 4 µmol/L ATO and 40 mmol/L Met alone and in combination, for 6 h. (E) Mitochondrial live staining with Mito tracker red after treatment of GFP-LC3-labeled HeLa cells with ATO and Met for 6 h, and observation of autophagic vesicle formation and co-localization of mitochondria and autophagosomes by laser confocal microscopy (as indicated with blue ▲) (original magnification, 40×).

Inhibition of HeLa cell proliferation by 40 mmol/L Met and 4 µmol/L ATO was accompanied by the formation of a numerous autophagosomes and mitochondrial autophagosomes in the cytoplasm (Figure 3A). The conversion of the cellular autophagy-related protein LC3-I to LC3-II increased, and expression of the autophagy-related protein Beclin1 also increased, accompanied by a decrease in the autophagy lysosomal substrate protein p62 (Figure 3B and C). Treatment with the combined drugs for 6 h further increased the intracellular conversion of LC3-I to LC3-II and the level of Beclin1 (Figure 3D), indicating that Met enhanced the autophagy-inducing effect of ATO on HeLa cells.

**Figure 4:**
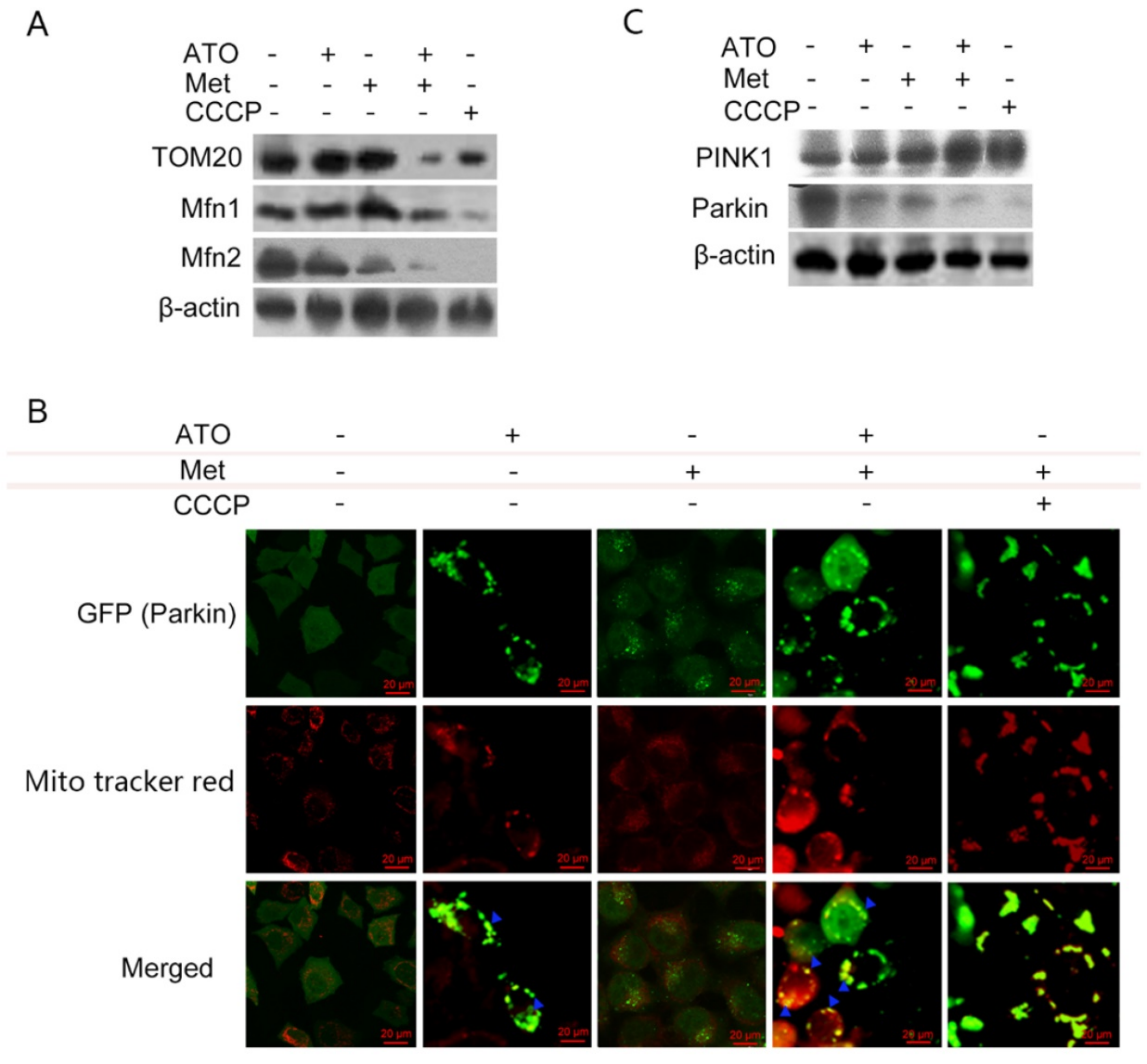
PINK1/Parkin pathway is involved in ATO- and Met-induced mitochondrial mitophagy in HeLa cells. ATO 4 µmol/L and Met 40 mmol/L were applied to HeLa cells for 6 h, with the mitochondrial uncoupling agent CCCP (Sigma ‑Aldrich, Merck KGaA, Darmstadt, Germany) as a positive control. (A) Changes in mitochondrial mitophagy-related proteins; (B) co-localization of Parkin and mitochondria after ATO and Met treatment of GFP-Parkin-labeled HeLa cells for 6 h, using Mito tracker red live mitochondrial staining and fluorescence microscopy (as indicated with blue ▲) (original magnification, 40×). (C) Effects of above treatments on intracellular PINK1 and Parkin proteins.

Under normal conditions, LC3-I was distributed diffusely in the cytoplasm, and was converted to LC3-II and accumulated on the autophagosome membrane during autophagy. Treatment of GFP-LC3-labeled HeLa cells with 40 mmol/L Met and 4 µmol/L ATO alone or in combination for 6 h resulted in the accumulation of numerous green fluorescent autophagic vesicles in the cytoplasm, as shown by laser confocal microscopy, compared with a uniform distribution of GFP throughout the untreated cells. We labelled mitochondria with Mito tracker red, and showed that mitochondrial red fluorescence was uniformly distributed throughout the cytoplasm in untreated cells, but was clustered in strips and co-localized with autophagosomes in treated cells (Figure 3E). Levels of the mitochondrial membrane protein TOM20 and the mitochondrial fusion proteins Mfn 1 and Mfn 2 were also significantly reduced in HeLa cells. Combined treatment with ATO and Met had a synergistic effect (Figure 4A), suggesting that ATO promoted Met-induced mitochondrial autophagy in HeLa cells. Furthermore, the Met-enhanced ATO induced mitophagy was closely consistent with the proliferation-inhibition of HeLa cells (Figures 1B,1C), and with the occurrence and development of mitophagy the cells showed significant apoptotic death (Figures 2A,2B). These mean that Met synergizes ATO triggering mitophagic cell death in HeLa cells.

### Pink1/Parkin pathway mediates Met- and ATO-induced mitophagy and mitophagic death in HeLa cells

The translocase TOM20 is a key receptor on the outer mitochondrial membrane, which recognizes the mitochondrial preprotein, maintains the unfolded state of the pre-substrate protein, and prevents protein aggregation in the mitochondria. Mfn1 and Mfn2 on the outer mitochondrial membrane play important roles at different stages in the process of mitochondrial fusion: Mfn1 promotes the binding of mitochondria to each other in the early stage of fusion, while Mfn2 mainly promotes endosomal docking in the late stage of mitochondrial fusion. Treatment of HeLa cells with ATO and Met separately for 6 h had no significant effect on TOM20 expression, but treatment with the combination of both drugs resulted in a significant downregulation of TOM20, accompanied by significant downregulation of both Mfn1 and Mfn2 (Figure 4A). These results suggested that the combination of ATO and Met caused mitophagic mitochondrial degradation in HeLa cells.

When cells are damaged, PINK1 accumulates stably on the outer mitochondrial membrane surface and senses a decrease in mitochondrial membrane potential. It then recruits parkin to translocate to the damaged mitochondria, mediating the onset of mitochondrial autophagy. ATO- and Met-treated GFP-Parkin-labeled HeLa cells showed granular orange fluorescence after co-localization with GFP-Parkin and the mitochondrial activity probe Mito tracker red under fluorescence microscopy, indicating that Parkin translocated to the mitochondrial membrane and participated in mitochondrial autophagy after mitochondrial damage (Figure 4B).

Treatment of HeLa cells with 40 mmol/L Met and 4 µmol/L ATO separately for 6 h resulted in a significant increase in levels of PINK1, accompanied by a decrease in Parkin levels. These changes were more significant after combined treatment with ATO and Met (Figure 4C), demonstrating that the Pink1-Parkin pathway was involved in Met-enhanced, ATO-induced mitophagy and autophagic apoptosis in HeLa cells.

## Discussion

In addition to its hypoglycemic effect, Met is also effective in reducing the risk of cardiovascular disease, protecting nerve cells, improving immunity, and inhibiting the development of tumor cells 1,31-34. Diabetic patients treated with Met also showed relatively lower tumor morbidity and mortality compared with those not treated with Met 35. Met also exhibits different regulatory effect on mitophagy in different tumor cell lines 2,3. It can suppress tumor proliferation by activating AMPK and inhibiting mTOR, inducing mitophagy formation, and enhancing tumor sensitivity to chemotherapeutic agents 12. Some novel regulatory mechanisms have been identified, and Met was shown to downregulate signaling and transcriptional activator 3 to cause an increase in tumor mitophagy and apoptosis levels in esophageal squamous epithelial cells. However, Met inhibited autophagy in some tumor cell lines 36. In the current study, Met inhibited the proliferative activity of cervical cancer HeLa cells in a time-concentration-dependent manner, accompanied by a significant increase in apoptosis, demonstrating that Met can inhibit cell proliferation by inducing apoptosis in HeLa cells. Although the ability of Met to reduce the incidence of malignant tumors in patients with type II diabetes has made it a hot topic of antitumor research in recent years, there is still no conclusive evidence regarding the antitumor properties and mechanism of Met, thus limiting its use in clinical tumor therapy.

ATO has recently been used widely for the treatment of hematologic tumors and a variety of solid tumors, with good results 26,27,37,38. Numerous studies have demonstrated that ATO induces apoptosis in tumor cells, often accompanied by autophagy 39. Some studies also suggested that ATO not only induced apoptosis in cervical cancer cells, but also increased their sensitivity to radiation 40. The present results demonstrated that some concentrations of ATO also inhibited the proliferative activity of HeLa cells in a time-concentration-dependent manner. Moreover, 2 and 4 µmol/L ATO increased the apoptosis rate of HeLa cells, resulting in morphological changes such as dense chromatin, border set, nuclear fragmentation and apoptotic vesicle formation under both light and transmission electron microscopy. These results suggest that certain concentrations of ATO can effectively induce apoptosis in cervical cancer HeLa cells.

Treatment of HeLa cells with Met and ATO alone and in combination induced apoptosis. Meanwhile, the autophagy phenomenon was accompanied by an increase in the conversion of LC3-I to LC3-II, increases in the mitophagy-related protein Beclin1 and the number of autophagosomes, and a decrease in the autophagy lysosomal substrate protein p62. Moreover, these changes were more pronounced after treatment with the combination of Met and ATO, indicating that ATO promoted the apoptotic effects of Met in HeLa cells and enhanced the occurrence of autophagy.

Autophagy is a process of organelle or abnormal protein degradation mediated by intracellular lysosomes, and is necessary for cell survival and maintaining the stability of the internal environment under various stresses 41. Mitochondria are important sites of autophagy in cells. When cells are stimulated, mitochondria are damaged and depolarized, and the damaged mitochondria are wrapped into autophagosomes and fused with lysosomes to degrade the damaged mitochondria, referred to as mitochondrial mitophagy 41,42. Impairment of this process may lead to cell death, and excessive activation of autophagy removes important organelles, including mitochondria, from the cell, thereby causing cell damage and eventually leading to mitophagic cell death 42,43. In addition to mitophagic degradation, many damaged mitochondrial and outer mitochondrial membrane and interstitial proteins are degraded by the PINK1/Parkin signaling pathway, and degradation of the inner mitochondrial membrane and matrix proteins is mostly mediated by Parkin-dependent mitochondrial autophagy 44,45. The mitochondrial outer membrane protein PINK1 is a molecular receptor for damaged mitochondria. Parkin protein has E3 ubiquitin-protein ligase activity, and its main function is to mediate the ubiquitination of substrates, regulate protein degradation, and signal transduction 46. PINK1 and Parkin are widely expressed in various tissues and organs, and are believed to act together to play a crucial role as the first line of mitochondrial defense. PINK1 is located upstream of Parkin in the same gene signaling pathway, and they interact with each other to mediate the degradation of damaged mitochondria via the autophagy-lysosome pathway 43,44. Under normal conditions, Parkin is mostly localized in the cytosol. When intracellular PINK1 senses a stimulus that causes mitochondrial depolarization, Parkin translocates from the cytosol to the mitochondria and activates autophagy to clear the damaged mitochondria; however, the exact mechanism is unknown. Both ATO and Met, alone or in combination, induced the formation of mitochondrial autophagosomes in HeLa cells, accompanied by a decrease in Parkin protein levels and an increase in PINK1 expression, suggesting that stimulation by both drugs caused intracellular PINK1 to accumulate on the outer mitochondrial membrane surface to sense the decrease in mitochondrial membrane potential, and recruit Parkin to translocate to the damaged mitochondria. This in turn triggered mitochondrial autophagy to clear the damaged mitochondria, resulting in a decrease in Parkin levels, accompanied by activation of the apoptosis-related proteins Bax/Bcl-2 such as mitochondrial fusion proteins Mfn1 and Mfn2 ubiquitination, which induced mitochondrial autophagic apoptosis in cells. Moreover, the above mitochondrial mitophagic effect was stronger after combined treatment with both drugs.

Mitochondria act as an intracellular energy supply center and can initiate apoptosis through mitochondrial signaling. Most proteins need to be transported into the mitochondria by the TOM system of mitochondrial outer membrane translocases in order to act 47. TOM20 is one of the key receptors on the outer mitochondrial membrane that recognizes the mitochondrial precursor peptide and maintains the unfolded state of the pre-substrate protein to prevent protein aggregation in the mitochondria 48,49. Mitochondria maintain a dynamic balance of fusion and division to ensure mitochondrial physiological function and normal morphology 47. Mfn1 and Mfn2 on the outer mitochondrial membrane each play an important role in the mitochondrial fusion process in different stages of fusion 50. Mfn1 promotes the binding of mitochondria to each other in the early stage of fusion, while Mfn2 mainly plays a role in the late stage of mitochondrial fusion, by promoting inner membrane docking. ATO and Met induced autophagy in HeLa cells, while levels of TOM20 and the mitochondrial fusion proteins Mfn1 and Mfn2 were significantly reduced, and this effect was more obvious after treatment with the combination of the two drugs.

Numerous studies have demonstrated that Met inhibits the proliferation of tumor cells but has no effect on normal cells. It has also been shown to reduce the risk of cardiovascular disease, protect neural cells, and improve immunity. In contrast, ATO is a more commonly used and effective chemotherapeutic agent for the treatment of leukemia, but its side effects are greater. The combination of the advantages of both drugs could thus improve the clinical efficacy and the quality of survival in cancer patients.

The present study showed that Met enhanced the effects of ATO to activate Bax/Bcl-2-related proteins, and to inhibit the proliferation and induce apoptosis in HeLa cells. Met also enhanced ATO induced the accumulation of autophagosomes in HeLa cells, increased the conversion of LC3-I to LC3-II, and decreased levels of the autophagy lysosomal substrate protein p62, as well as decreasing the mitochondrial membrane potential and activating the mitochondrial translocase TOM system and PINK1/Parkin signaling pathway to activate mitophagy, leading to mitophagic apoptosis. The above results suggest that metformin synergizes arsenic trioxide to trigger Parkin/pink1-dependent mitophagic cell death in HeLa cells, which show not only theoretical significance of exploring anti-cancer mechanisms of metformin but also the potential application of combination of metformin and arsenic trioxide in treatment of cervical cancer.

## References

[1] Wang YW, He SJ, Feng X, Cheng J, Luo YT, Tian L, Huang Q (2017). Metformin: a review of its potential indications. Drug Des Devel Ther.

[2] Cha JH, Yang WH, Xia W, Wei Y, Chan LC, Lim SO, Li CW, Kim T, Chang SS, Lee HH, Hsu JL, Wang HL, Kuo CW, Chang WC, Hadad S, Purdie CA, McCoy AM, Cai S, Tu Y, Litton JK, Mittendorf EA, Moulder SL, Symmans WF, Thompson AM, Piwnica-Worms H, Chen CH, Khoo KH, Hung MC (2018). Metformin promotes antitumor immunity via endoplasmic-reticulum-associated degradation of PD-L1. Mol Cell.

[3] Wang C, Yang Y, Zhang Y, Liu J, Yao Z, Zhang C (2019). Protective effects of metformin against osteoarthritis through upregulation of SIRT3-mediated PINK1/Parkin-dependent mitophagy in primary chondrocytes. Biosci Trends.

[4] Lee J, Yesilkanal AE, Wynne JP, Frankenberger C, Liu J, Yan J, Elbaz M, Rabe DC, Rustandy FD, Tiwari P, Grossman EA, Hart PC, Kang C, Sanderson SM, Andrade J, Nomura DK, Bonini MG, Locasale JW, Rosner MR (2019). Effective breast cancer combination therapy targeting BACH1 and mitochondrial metabolism. Nature.

[5] Faria J, Negalha G, Azevedo A, Martel F (2019). Metformin and breast cancer: molecular targets. J Mammary Gland Biol Neoplasia.

[6] Sacco F, Silvestri A, Posca D, Pirrò S, Gherardini PF, Castagnoli L, Mann M, Cesareni G (2016). Deep proteomics of breast cancer cells reveals that metformin rewires signaling networks away from a pro-growth state. Cell Syst.

[7] Coyle C, Cafferty FH, Vale C, Langley RE (2016). Metformin as an adjuvant treatment for cancer: a systematic review and meta-analysis. Ann Oncol.

[8] Dankner R, Agay N, Olmer L, Murad H, Keinan Boker L, Balicer RD, Freedman LS (2019). Metformin treatment and cancer risk: cox regression analysis, with time-dependent covariates, of 320,000 persons with incident diabetes mellitus. Am J Epidemiol.

[9] De Souza A, Khawaja KI, Masud F, Saif MW (2016). Metformin and pancreatic cancer: Is there a role?. Cancer Chemother Pharmacol.

[10] Wang JC, Li GY, Wang B, Han SX, Sun X, Jiang YN, Shen YW, Zhou C, Feng J, Lu SY, Liu JL, Wang MD, Liu PJ (2019). Metformin inhibits metastatic breast cancer progression and improves chemosensitivity by inducing vessel normalization via PDGF-B downregulation. J Exp Clin Cancer Res.

[11] Zhou HY, Yao XM, Chen XD, Tang JM, Qiao ZG, Wu XY (2019). Mechanism of metformin enhancing the sensitivity of human pancreatic cancer cells to gem-citabine by regulating the PI3K/Akt/mTOR signaling pathway. Eur Rev Med Pharmacol Sci.

[12] Wang Y, An H, Liu T, Qin C, Sesaki H, Guo S, Radovick S, Hussain M, Maheshwari A, Wondisford FE, O'Rourke B, He L (2019). Metformin improves mitochondrial respiratory activity through activation of AMPK. Cell Rep.

[13] Wang Z, Guo J, Han X, Xue M, Wang W, Mi L, Sheng Y, Ma C, Wu J, Wu X (2019). Metformin represses the pathophysiology of AAA by suppressing the activation of PI3K/AKT/mTOR/autophagy pathway in ApoE(-/-) mice. Cell Biosci.

[14] Roncolato F, Lindemann K, Willson ML, Martyn J, Mileshkin L (2019). PI3K/AKT/mTOR inhibitors for advanced or recurrent endometrial cancer. Cochrane Database Syst Rev.

[15] Jun KH, Lee JE, Kim SH, Jung JH, Choi HJ, Kim YI, Chin HM, Yang SH (2015). Clinicopathological significance of N-cadherin and VEGF in advanced gastric cancer brain metastasis and the effects of metformin in preclinical models. Oncol Rep.

[16] Najbauer J, Kraljik N, Németh P (2014). Glioma stem cells: markers, hallmarks and therapeutic targeting by metformin. Pathol Oncol Res.

[17] Porporato PE, Filigheddu N, Pedro JMB, Kroemer G, Galluzzi L (2018). Mitochondrial metabolism and cancer. Cell Res.

[18] Baechler BL, Bloemberg D, Quadrilatero J (2019). Mitophagy regulates mitochondrial network signaling, oxidative stress, and apoptosis during myoblast differentiation. Autophagy.

[19] Wada J, Nakatsuka A (2016). Mitochondrial dynamics and mitochondrial dysfunction in diabetes. Acta Med Okayama.

[20] Foretz M, Hébrard S, Leclerc J, Zarrinpashneh E, Soty M, Mithieux G, Sakamoto K, Andreelli F, Viollet B (2010). Metformin inhibits hepatic gluconeogenesis in mice independently of the LKB1/AMPK pathway via a decrease in hepatic energy state. J Clin Invest.

[21] Cui Y, Zhou J, Rong F (2020). Combination of metformin and RG7388 enhances inhibition of growth and induction of apoptosis of ovarian cancer cells through the PI3K/AKT/mTOR pathway. Biochem Biophys Res Commun.

[22] Sun R, Zhai R, Ma C, Miao W (2020). Combination of aloin and metformin enhances the antitumor effect by inhibiting the growth and invasion and inducing apoptosis and autophagy in hepatocellular carcinoma through PI3K/AKT/mTOR pathway. Cancer Med.

[23] Kim HG, Hien TT, Han EH, Hwang YP, Choi JH, Kang KW, Kwon K, Kim BH, Kim SK, Song GY, Jeong TC, Jeong HG (2011). Metformin inhibits P-glycoprotein expression via the NF-κB pathway and CRE transcriptional activity through AMPK activation. Br J Pharmacol.

[24] Karnevi E, Said K, Andersson R, Rosendahl AH (2013). Metformin-mediated growth inhibition involves suppression of the IGF-I receptor signalling pathway in human pancreatic cancer cells. BMC Cancer.

[25] Quinn BJ, Dallos M, Kitagawa H, Kunnumakkara AB, Memmott RM, Hollander MC, Gills JJ, Dennis PA (2013). Inhibition of lung tumorigenesis by metformin is associated with decreased plasma IGF-I and diminished receptor tyrosine kinase signaling. Cancer Prev Res (Phila).

[26] Kasukabe T, Okabe-Kado J, Kato N, Honma Y, Kumakura S (2015). Cotylenin A and arsenic trioxide cooperatively suppress cell proliferation and cell invasion activity in human breast cancer cells. Int J Oncol.

[27] Kim JH, Kim JH, Yu YS, Kim DH, Kim CJ, Kim KW (2009). Antitumor activity of arsenic trioxide on retinoblastoma: cell differentiation and apoptosis depending on arsenic trioxide concentration. Invest Ophthalmol Vis Sci.

[28] Xu C, Wang X, Zhou Y, Chen FX, Wang H, Li K, Fan H, Tang X, Jiang G, Zhang J (2019). Synergy between arsenic trioxide and JQ1 on autophagy in pancreatic cancer. Oncogene.

[29] Torre LA, Bray F Siegel RL, Ferlay J Lortet-Tieulent J, Jemal A Global cancer statistics, Ca-Cancer J Clin. 2015; 65: 87-108.

[30] Aniogo EC, George BPA, Abrahamse H (2020). Role of Bcl-2 family proteins in photodynamic therapy mediated cell survival and regulation. Molecules.

[31] Markowicz-Piasecka M, Sikora J, Szydłowska A, Skupień A, Mikiciuk-Olasik E, Huttunen KM (2017). metformin - a future therapy for neurodegenerative diseases: theme: drug discovery, development and delivery in alzheimer's disease guest Editor: davide brambilla. Pharm Res.

[32] Nesti L, Natali A (2017). Metformin effects on the heart and the cardiovascular system: A review of experimental and clinical data. Nutr Metab Cardiovasc Dis.

[33] Ma R, Yi B, Riker AI, Xi Y (2020). Metformin and cancer immunity. Acta Pharmacol Sin.

[34] Schulten HJ (2018). Pleiotropic Effects of Metformin on Cancer. Int J Mol Sci.

[35] Rizos CV, Elisaf MS (2013). Metformin and cancer. Eur J Pharmacol.

[36] Feng Y, Ke C, Tang Q, Dong H, Zheng X, Lin W, Ke J, Huang J, Yeung S-C J, Zhang H (2014). Metformin promotes autophagy and apoptosis in esophageal squamous cell carcinoma by downregulating Stat3 signaling. Cell Death Dis.

[37] Chen Jing, Cheng Jie, Yi Juan, Xie Bei, Lin Li, Liu Zhuan, Zhao Huaishun, Wang Bei, Ai Ziying, Yue Yang, Wei Hulai (2016). Differential expression and response to arsenic stress of MRPs and ASAN1 determine sensitivity of classical multidrug-resistant leukemia cells to arsenic trioxide. Leuk Res.

[38] Jing Chen, Baoying Tian, Cunmin Zhou, Jingjing Sun, Li Lin, Shucheng Jin, Quanrui Liu, Siyu Fu, Lian Liu, Hang Liu, Zhewen Zhang, Caili Li, Hulai Wei (2019). A novel resveratrol-arsenic trioxide combination treatment synergistically induces apoptosis of adriamycin-selected drug-resistant leukemia K562 cells. Journal of Cancer.

[39] Li CL, Wei Hulai, Chen J, Wang B, Xie B, Fan LL, Li LJ (2014). Arsenic trioxide induces autophagy and antitumor effects in Burkitt's lymphoma Raji cells. Oncol Rep.

[40] Young-Hee Kang, Su-Jae Lee (2008). Role of p38 MAPK and JNK in enhanced cervical cancer cell killing by the combination of arsenic trioxide and ionizing radiation. Oncol Rep.

[41] Glick D, Barth S, Macleod KF (2010). Autophagy: cellular and molecular mechanisms. J Pathol.

[42] Yoo SM, Jung YK (2018). A molecular approach to mitophagy and mitochondrial dynamics. Mol Cells.

[43] Pickles S, Vigié P, Youle RJ (2018). Mitophagy and quality control mechanisms in mitochondrial maintenance. Curr Biol.

[44] Pickrell AM, Youle RJ (2015). The roles of PINK1, parkin, and mitochondrial fidelity in Parkinson's disease. Neuron.

[45] Ivankovic D, Chau KY, Schapira AH, Gegg ME (2016). Mitochondrial and lysosomal biogenesis are activated following PINK1/parkin-mediated mitophagy. J Neurochem.

[46] Sliter DA, Martinez J, Hao L, Chen X, Sun N, Fischer TD, Burman JL, Li Y, Zhang Z, Narendra DP, Cai H, Borsche M, Klein C, Youle RJ (2018). Parkin and PINK1 mitigate STING-induced inflammation. Nature.

[47] Bausewein T, Naveed H, Liang J, Nussberger S (2020). The structure of the TOM core complex in the mitochondrial outer membrane. Biol Chem.

[48] Di Maio R, Barrett PJ, Hoffman EK, Barrett CW, Zharikov A, Borah A, Hu X, McCoy J, Chu CT, Burton EA, Hastings TG, Greenamyre JT (2016). alpha-Synuclein binds to TOM20 and inhibits mitochondrial protein import in Parkinson's disease. Sci Transl Med.

[49] Eliyahu E, Pnueli L, Melamed D, Scherrer T, Gerber AP, Pines O, Rapaport D, Arava Y (2010). Tom20 mediates localization of mRNAs to mitochondria in a translation-dependent manner. Mol Cell Biol.

[50] Chen H, Detmer SA, Ewald AJ, Griffin EE, Fraser SE, Chan DC (2003). Mitofusins Mfn1 and Mfn2 coordinately regulate mitochondrial fusion and are essential for embryonic development. J Cell Biol.

